# Editorial: Immunomodulatory Approaches in Cardiovascular Diseases

**DOI:** 10.3389/fcvm.2022.873452

**Published:** 2022-03-31

**Authors:** George W. Booz, Raffaele Altara, Fouad A. Zouein

**Affiliations:** ^1^Department of Pharmacology and Toxicology, School of Medicine, University of Mississippi Medical Center, Jackson, MS, United States; ^2^Department of Pathology, School of Medicine, University of Mississippi Medical Center, Jackson, MS, United States; ^3^Department of Pharmacology and Toxicology, Faculty of Medicine, American University of Beirut Medical Center, Beirut, Lebanon; ^4^The Cardiovascular, Renal, and Metabolic Diseases Research Center of Excellence, American University of Beirut Medical Center, Beirut, Lebanon

**Keywords:** immunomodulation, cardiovascular diseases, immune system, cardiac remodeling, cardiorenal, vasculitis, immunotherapies

A revolution has unfolded in our midst. Over the last two decades or so, a substantial body of evidence has developed showing that the immune and cardiovascular systems are intertwined. There are two aspects to this revolution. First, it is by now firmly established that the immune system contributes to the progression of cardiovascular disease (CVD), atherosclerosis in particular, although the intricacies are not fully understood. Nor do we understand how the immune system affects the initiation and progression of various cardiac conditions or myopathies. The second aspect is that the immune system can be engaged to target CVD. For instance, proof of concept evidence was recently presented that cardiac fibrosis can be attenuated by immunotherapy, involving adoptive transfer of CAR T cells expressing a cognate T cell receptor against fibroblast activation protein (FAP) (1). A subsequent study provides further evidence to support the therapeutic feasibility of this approach to counter maladaptive cardiac remodeling (2). In this study, T cell–targeted lipid nanoparticles were used to produce transient antifibrotic CAR T cells *in vivo*, which in a mouse model of heart injury, reduced fibrosis and restored cardiac function.

To celebrate this revolution, Frontiers in Cardiovascular Medicine presents the series entitled *Immunomodulatory Approaches in Cardiovascular Diseases*. Immunomodulatory approaches can be defined as “all interventions that modulate and curb the immune response of the host rather than targeting the disease itself” (Ammar et al.). This collection of 11 articles covers a broad swath, including reviews and original research articles, preclinical studies, a clinical trial, and case reports. They cover such topics as cardiovascular and metabolic diseases, cardiorenal syndromes, atrial fibrillation, Graves' disease, cardiac sarcoidosis, COVID-19, stress-induced myocardial remodeling, and dilated cardiomyopathy (Figure 1).

**Figure 1:**
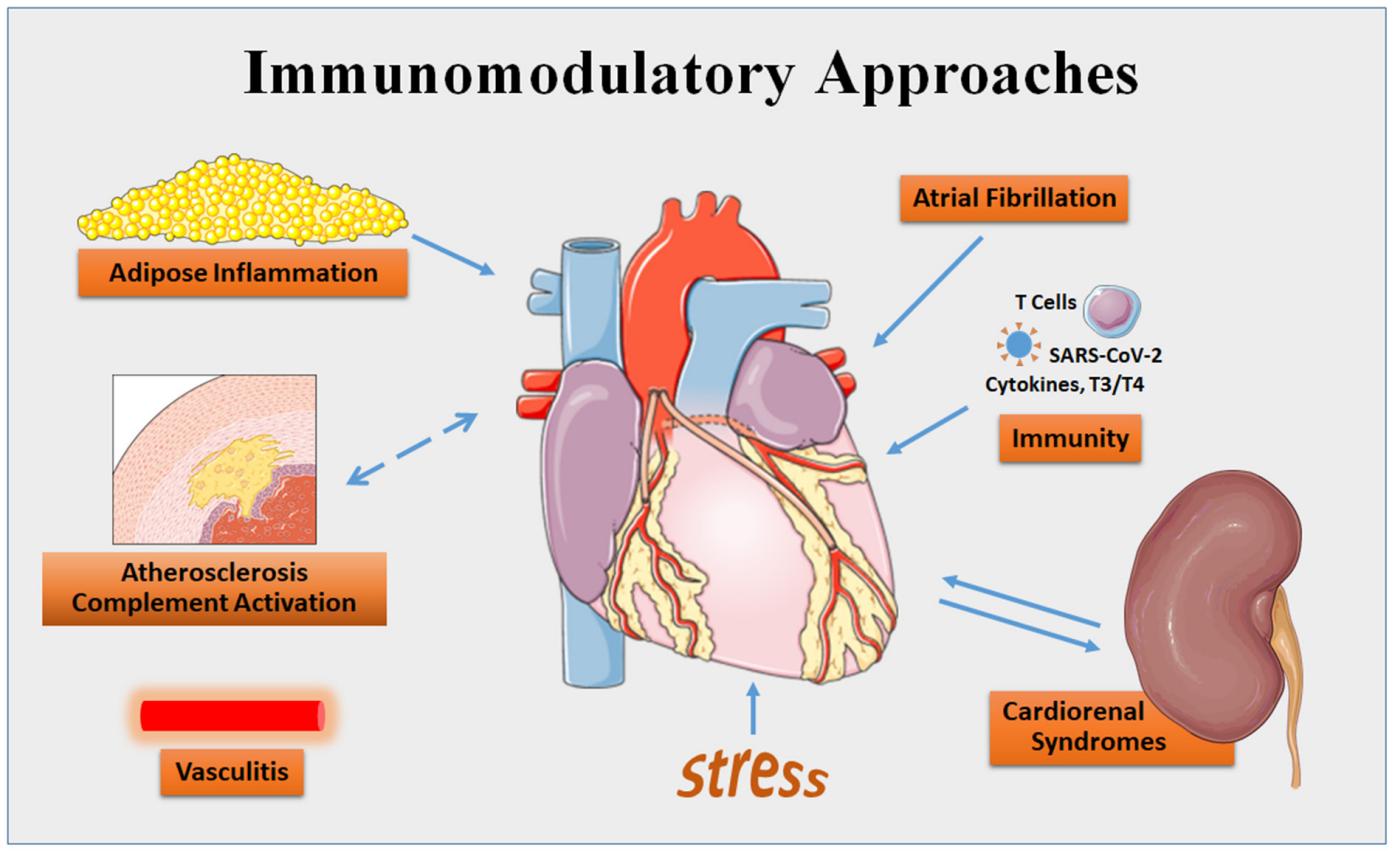
Areas of cardiovascular disease targeted by immunomodulatory approaches that are covered by the series in Frontiers in Cardiovascular Medicine. Some content is adapted from Servier Medical Art (https://smart.servier.com/).

A comprehensive review by AlZaim et al. tackles the topic of adipose tissue immunomodulation and its impact on CVDs. This impact varies according to the type of adipose tissue depot. Significant crosstalk occurs among adipocytes, adipokines, and both resident and infiltrating cells of the innate and adaptive immune systems. As discussed, immune cell and adipokine profile dysfunction underpins adipose tissue inflammation, which in turn influences the heart and vasculature. The authors provide an overview of immunomodulatory approaches targeted toward adipose tissue for treating metabolic disorders and CVDs, including exercise and lifestyle modifications and anti-diabetic drugs, as well as several novel approaches, such as those that alter the gut microbiota. Another review article, deals with immunomodulatory approaches in diabetes-induced cardiorenal syndromes (Ammar et al.). After a brief introduction to immunomodulatory approaches in diabetic cardiomyopathy and nephropathy, this comprehensive article reviews the epidemiology and classifications of cardiorenal syndrome, which denotes the confluence of heart-kidney relationships such that dysfunction of one may initiate disease in the other via common neurohormonal, hemodynamic, biochemical, and/or immunological feedback pathways. The authors focus in on therapeutic approaches that target the immunomodulatory pathways implicated in diabetes-induced cardiorenal syndrome, namely the renin-angiotensin system, JAK/STAT signaling, and oxidative stress.

Two case-based reviews highlight the impact of the immune system on the heart and/or vascular system and the therapeutic importance of targeting it in the real world. One article deals with treatment of autoimmune myocarditis in Graves' disease, which is an immune system disorder that results in overproduction of thyroid hormones (hyperthyroidism) (Wu et al.). A second article presents a case series reporting the successful treatment of cardiac sarcoidosis with tumor necrosis factor alpha (TNFα) antagonists (Stievenart et al.). This life-threatening disease, which lacks clear recommendations for treatment, is a rare inflammatory condition in which clusters of white blood cells form granulomas in different areas of the heart, which may disrupt heart rhythm, blood flow, and normal heart function. Apropos of the present COVID-19 pandemic, an article by Zareef et al. addresses the course of SARS-CoV-2–induced illness in pediatric patients, both otherwise healthy children and those with congenital heart disease. Consequences include myocarditis, cardiogenic shock, arrhythmias, and multisystem inflammatory syndrome. Another original research study by Itani et al. presents data supporting the hypothesis that the inflammatory immune response is exacerbated in patients with both atrial fibrillation and cardiometabolic syndrome compared to either condition alone. They examined inflammatory cytokines and fibrotic markers as well as cytokine genetic profiles. Their approach has importance for the development of novel immunomodulatory therapeutic strategies for treating atrial fibrillation.

Two articles in the series deal with atherosclerosis. One gene expression-based bioinformatics study provides a comprehensive analysis of the immune cell infiltrates and aberrant pathway activation in atherosclerotic plaque (Han et al.). The findings support the idea that patients with coronary artery disease have an inflammatory immune microenvironment, which may be responsive to anti-inflammatory therapies under investigation. A preclinical study in Apoe^−/−^ mice examined the therapeutic possibility of targeting complement activation in atherosclerosis (Dai et al.). Complement activation has been implicated in the development of atherosclerosis, but how that occurs is not known. The present study was based upon the observation that in ischemia-reperfusion injury, ischemia induces exposure of neoepitopes recognized by natural self-reactive IgM antibodies, which in turn activate complement. The authors used a novel construct (C2scFv-Crry), consisting of a single chain antibody (scFv) linked to a complement inhibitor (Crry) that functions at C3 activation. The scFv moiety was derived from C2 IgM mAb, which recognizes phospholipid neoepitopes known to be expressed after ischemia. In Apoe^−/−^ mice fed a high-fat diet, C2scFv-Crry administration decreased atherosclerotic plaque in the aorta and aortic root, reduced deposition of endogenous total IgM in the plaque, decreased lipid content in the lesion, and reduced serum oxLDL levels. Thus, neoepitope targeted complement inhibitors may be a novel therapeutic strategy to combat atherosclerosis. Somewhat related, leukocytoclastic vasculitis is a systemic autoimmune disease that is characterized by inflammation of the vascular endothelium and includes cutaneous small vessel vasculitis (CSVV) and anti-neutrophil cytoplasmic antibody-associated vasculitis (AAV). Risk haplotypes, genetic variants, susceptibility loci and pathways associated with vasculitis immunopathogenesis have been identified and have laid the foundation for personalized medicine with targeted therapies. A review article by Yap et al. discusses pathways involved in disease pathogenesis and the underlying genetic associations in different populations worldwide. As noted, specific genetic variants predisposing individuals to CSVV and their pathogenic mechanisms are incompletely defined. Determining the immunopathogenic pathways in vasculitis and associated genetic variations will enable development of targeted personalized therapies.

Stress, including the psychological pressures of daily life, can adversely affect the structure and function of the heart, for example takotsubo cardiomyopathy. A study in the series examined maladaptive cardiac remodeling in an isoproterenol-induced cardiomyopathy mouse model (Adzika et al.). The authors found that amlexanox, an anti-inflammatory and immunomodulatory drug, attenuated the myocardial hypertrophy, fibrosis, and inflammation seen with isoproterenol. These actions were enhanced by the adenylyl cyclase activator, forskolin. In isolated macrophages, amlexanox acted by inhibiting a GRK5-mediated proinflammatory effect and, along with forskolin, facilitating cAMP-mediated immunoregulation. Finally, dilated cardiomyopathy, characterized by dilation and systolic dysfunction of one or both ventricles, may have a genetic basis or occur due to various etiologies that cause myocardium inflammation. New insights provide a better understanding of the pathogenesis of dilated cardiomyopathy by linking the genetic and inflammatory causes together. A review by Kadhi et al. summarizes the genetic and inflammatory causes underlying dilated cardiomyopathy and the pathways amenable to immunomodulatory strategies to salvage and prevent heart failure linked to the disease.

In recent years, we have made much progress in understanding the interplay between the immune system and CVDs. We stand on the cusp of another revolution that implements this knowledge into novel therapeutic approaches, possibly in the context of personalized medicine. The exciting articles in *Immunomodulatory Approaches in Cardiovascular Diseases* should attract much attention and foster further investigations to advance medical knowledge.

## Author Contributions

GB and FZ helped edit the text. All authors contributed to the inception and writing of the manuscript. All authors contributed to the article and approved the submitted version.

## Funding

This work was supported by grants to FZ from the American University of Beirut Faculty of Medicine [Grant Number MPP – 320145/320095 and URB – 103949] and by Center National de la Recherche Scientifique (CNRS) [Grant Number 103507/103487/103941/103944], Collaborative Research Stimulus (CRS) [Grant Number 103556], and Agence Nationale de la Recherche (ANR) [Grant – ANICOV-HF].

## Conflict of Interest

The authors declare that the research was conducted in the absence of any commercial or financial relationships that could be construed as a potential conflict of interest.

## Publisher's Note

All claims expressed in this article are solely those of the authors and do not necessarily represent those of their affiliated organizations, or those of the publisher, the editors and the reviewers. Any product that may be evaluated in this article, or claim that may be made by its manufacturer, is not guaranteed or endorsed by the publisher.
